# Impact of electronic AKI alert/care bundle on AKI inpatient outcomes: a retrospective single-center cohort study

**DOI:** 10.1080/0886022X.2024.2313177

**Published:** 2024-02-12

**Authors:** Michael Chen-Xu, Christopher Kassam, Emma Cameron, Szymon Ryba, Vivian Yiu

**Affiliations:** a West Suffolk Hospital NHS Foundation Trust, Suffolk, UK; b Cambridge University Hospitals NHS Foundation Trust, Cambridge, UK

**Keywords:** Acute kidney injury, electronic alerting, care bundle, renal failure, quality improvement

## Abstract

**Background:**

Outcomes among acute kidney injury (AKI) patients are poor in United Kingdom (UK) hospitals, and electronic alerts and care bundles may improve them. We implemented such a system at West Suffolk Hospital (WSH) called the ‘AKI order set’. We aimed to assess its impact on all-cause mortality, length of stay (LOS) and renal function among AKI patients, and its utilization.

**Methods:**

Retrospective, single-center cohort study of patients ≥ 18 years old with AKI at WSH, a 430-bed general hospital serving a rural UK population of approximately 280,000. 7243 unique AKI events representing 5728 patients with full data were identified automatically from our electronic health record (EHR) between 02 September 2018 and 1 July 2021 (median age 78 years, 51% male). All-cause mortality, LOS and improvement in AKI stage, demographic and comorbidity data, medications and AKI order set use were automatically collected from the EHR.

**Results:**

The AKI order set was used in 9.8% of AKI events and was associated with 28% lower odds of all-cause mortality (multivariable odds ratio [OR] 0.72, 95% confidence interval [CI] 0.57–0.91). Median LOS was longer when the AKI order set was utilized than when not (11.8 versus 8.8 days, *p* < .001), but was independently associated with improvement in the AKI stage (28.9% versus 8.7%, *p* < .001; univariable OR 4.25, 95% CI 3.53–5.10, multivariable OR 4.27, 95% CI 3.54–5.14).

**Conclusions:**

AKI order set use led to improvements in all-cause mortality and renal function, but longer LOS, among AKI patients at WSH.

## Introduction

### Background/rationale

Acute kidney injury (AKI) is a common and serious problem among hospital inpatients worldwide [1,2]. In the UK, estimates of incidence amongst inpatients range from 13% to 25.4%, while mortality may reach 30% [3,4]. Inpatients with coronavirus disease 2019 (COVID-19) have consistently been found to have AKI incidence of >20%, and AKI is an independent risk factor for increased in-hospital mortality [5]. AKI is also associated with an increased risk of *de novo* chronic kidney disease (CKD) and cardiovascular disease [6,7]. Recognition and care of AKI in UK hospitals is poor, despite national guidelines and quality standards [8].

Over the past decade, the widespread adoption of electronic health records (EHRs) has led to international interest in the development of electronic detection systems, alerts and care bundles to improve AKI management [9–11]. The evidence on the effectiveness of these interventions is mixed, and it is difficult to draw overall conclusions due to variability in study design and outcome measures. Studies to date have typically demonstrated improvement in AKI detection and in process measures such as medication review, fluid balance review and specialist nephrology consult, but little impact on outcome measures such as mortality and progression of AKI [12–17]. A recent systematic review and meta-analysis found a 19% reduction in in-hospital mortality across studies with a before-and-after design, but no significant effect on mortality in randomized controlled trials [17]. As healthcare providers have transitioned toward electronic records, not all studies have fully integrated detection and alerting with treatment recommendations and links to the direct ordering of appropriate interventions [11,12,14,15]. A further systematic review suggested that electronic AKI detection and alerting was associated with significant improvement in both process measures (diagnostic and therapeutic interventions) and outcome measures (renal function and mortality) only if coupled with treatment recommendations [18]. However, a large before-and-after study of >64,000 patients with AKI subsequently demonstrated significant reductions in mortality, dialysis and length of hospital stay from an alerting system not linked to specific treatment recommendations [19]. In addition, utilization of care bundle ordering (rather than ordering of individual elements) has been shown to be poor [12]. Overall, while there are some signals that electronic AKI alerting may be effective in improving outcome and (more convincingly) process measures, further evidence is needed to confirm these signals and to identify which type of alerting and ordering systems are most strongly associated with improved outcomes.

In this article we report on a single-center quality improvement project undertaken at a UK hospital from 2018–21. The project aimed to measure the impact and uptake of an integrated electronic detection/alerting system and order set introduced in 2018, the ‘AKI order set’, which was designed on the basis of national guidelines and quality standards. Given the ongoing COVID-19 pandemic, we also sought to assess its impact on the contribution of AKI to COVID-19 mortality. Lastly, we aimed to improve the utilization of this order set.

## Materials and methods

### Study design, setting and participants

This retrospective cohort study was conducted at the West Suffolk Hospital (WSH), a 430-bed general hospital serving a predominantly rural and elderly UK population of approximately 280,000. Elderly patients are at increased risk of short- and long-term mortality following AKI and progression to chronic kidney disease (CKD) [7]. All patients aged ≥18 years old admitted with or who developed AKI from the introduction of the AKI order set on 2 September 2018 were included. AKI and its stages were defined by KDIGO (Kidney Disease Improving Global Outcomes) criteria [20], and inpatient AKI events meeting these criteria were automatically identified *via* our EHR, ECare (Cerner).

**Figure 1.**
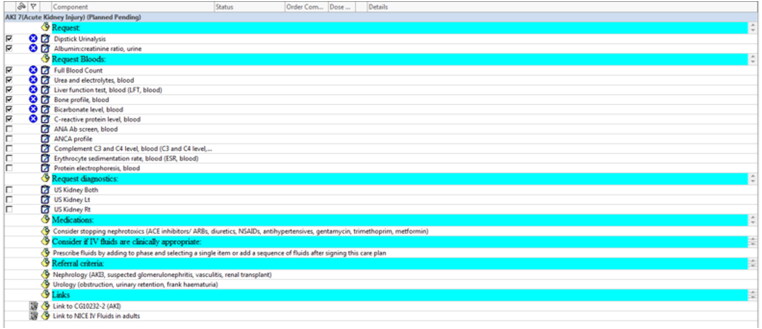
The electronic AKI care plan (AKI-CP). Checkboxes on the left when checked lead to the ordering of specific urine and/or blood tests, denoted by the ‘clipboard’ icon, part of the AKI-CP, e.g., ‘Dipstick Urinalysis’, or ‘Urea and electrolytes, blood’. Text to the right of the ‘pinned note’ icon represent specific advice relevant for the management of AKI part of the AKI-CP, e.g., ‘Consider stopping nephrotoxics (ACE inhibitors/ARBs, diuretics, NSAIDs, antihypertensives, gentamycin, trimethoprim, metformin)’. The text to the right of the ‘document’ and ‘pinned note’ icons under the ‘Links’ heading are links to the local and national guidelines for the management of AKI.

**Figure 2.**
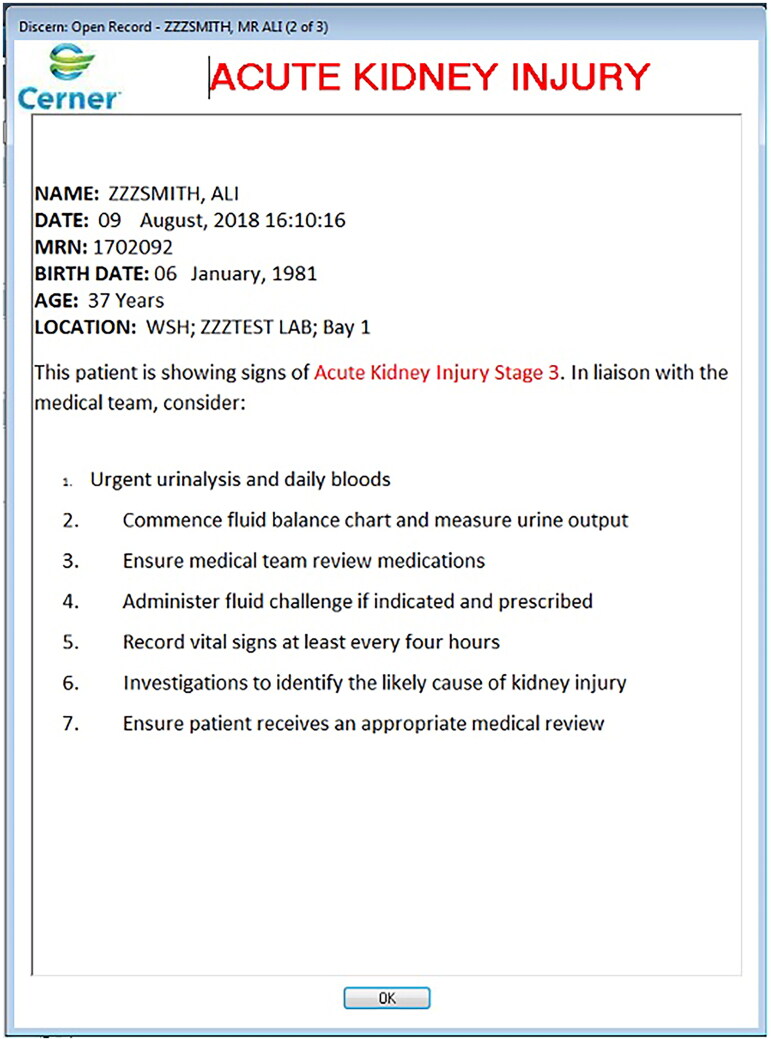
The interruptive electronic alert from the ‘AKI order set’. An example of the interruptive electronic alert from the ‘AKI order set’, which is triggered by AKI stage 2 or above. This alert provides information about the patient’s details, including their name, date of birth, age, location and Medical Records Number (MRN), and suggests that the use initiates the AKI-CP.

The ‘AKI order set’ consisted of an interruptive electronic alert, triggered when a user opened an EHR for a patient with AKI, and an ‘AKI care plan’ (AKI-CP) with recommended interventions (Figure 1). For medical practitioners, the alert would inform the user of the AKI stage and suggest AKI-CP initiation. For nursing staff, it would suggest interventions such as fluid balance monitoring and urinalysis (Figure 2). AKI-CP initiation allowed medical staff to request its elements, including links to recommended investigations and local guidelines. The design of the ‘AKI order set’ was influenced by the national guidelines and quality standards outlined in the National Confidential Enquiry into Patient Outcome and Death (NCEPOD) report [8]. Education about AKI recognition and management, including the use of the AKI order set, was provided to junior doctors annually through their mandatory education programme. We compared outcomes among AKI episodes that had the AKI-CP initiated with those that did not.

**Figure 3.**
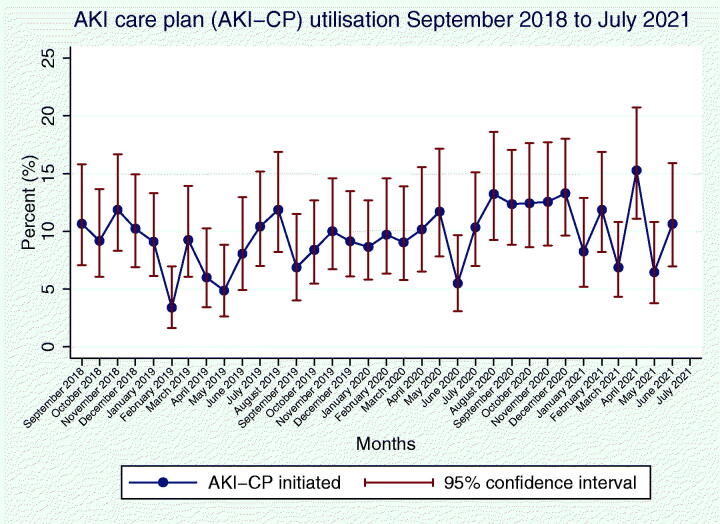
Utilization of the AKI care plan (AKI-CP) over time.

AKI order set utilization was measured both objectively, through an audit of electronic records, and subjectively, through a survey of junior doctors in October 2020 using Likert scales to quantify the self-reported frequency of order set requesting. Respondents were also asked about factors impeding order set utilization, and interventions which might increase utilization. Based on these responses, a quality improvement project was undertaken to increase utilization, consisting of educational sessions and posters. Subsequently both the audit and survey were repeated to measure the impact of these interventions on order set utilization (Figure 3).

### Covariate ascertainment

Demographic data including age, sex, type of admission, length of stay (LOS), discharge method/destination and date of death were obtained from our EHR on 14 September 2021. Comorbidity data were identified using the International Classification of Disease, Tenth Edition (ICD-10) codes, as documented on admission. Medications coded as prescribed during admission were automatically identified from the EHR, as was COVID-19 status, which was by detection of SARS-CoV-2 (Severe acute respiratory syndrome coronavirus 2) using reverse transcriptase polymerase chain reaction (RT-PCR) at the designated laboratory serving WSH.

### Outcome measures

The primary outcome was inpatient all-cause mortality. Secondary outcomes were LOS, improvement in the AKI stage, survival post discharge, the impact of COVID-19 infection among AKI patients on these, and the utilization of the AKI-CP.

### Ethical approval

After consulting the local Trust research team and reviewing local Trust policy, this study was considered exempt from the need for ethical approval as it was deemed to be a retrospective audit of anonymised data, collected routinely as part of a locally registered quality improvement project.

### Informed consent

As this study was a retrospective audit of routinely collected clinical data, it was deemed exempt from the requirement for informed consent.

### Statistical methods

Baseline characteristics were presented as mean (standard deviation), median (interquartile range [IQR]), or percentages for categorical variables as appropriate. Inpatient mortality and improvement in the AKI stage were analyzed using logistic regression, while LOS was analyzed using truncated negative binomial regression, both univariably and correcting for pre-specified confounders (age, sex, comorbidities, medications prescribed during admission, and first AKI stage) and COVID-19 status. Cox-proportional hazards ratios were analyzed at different timepoints due to several covariates violating the proportional hazards assumption, based on log-minus-log plots and scaled Schoenfeld residuals against time for categorical and numerical variables, respectively. Standardized survival curves incorporating time-dependent variables were also fitted using a flexible parametric approach, and restricted mean survival times (RMSTs) were calculated at different timepoints to further assess the impact of the AKI-CP on survival [21,22]. Given that the primary aim of the study was to assess the relationship between AKI order set utilization and the aforementioned outcomes of interest and pre-specified covariates were included based on their potential confounding effect(s), likelihood ratio testing for covariate selection and model fitting was not deemed necessary. Multiple imputation was not performed for missing data due to the low proportion of missing data. Two-sided *p* values <.05 were considered significant. For patients with multiple episodes of AKI, their first episode was considered as the index episode. All statistical analyses were conducted using Stata 15.1.

## Results

### Baseline characteristics

We identified 7274 unique AKI events in separate hospital admissions representing 5755 patients between 2 September 2018 and 1 July 2021. Data was missing from 34 AKI events representing 27 patients, leaving 7243 unique AKI events among 5728 patients in the complete case analysis. The median age was 78 (IQR 67–85) years and 51% were male (Table 1). The AKI-CP was initiated in 710/7243 AKI events (9.8%) from 577/5728 (10.1%) of patients. The most common comorbidities were diabetes (27.7%), heart failure (25.7%) and cancer (18.5%), followed by CKD (12.6%).

**Table 1.** Baseline characteristics of patients by AKI-CP initiation status.

	AKI-CP not initiated	AKI-CP initiated	Total
Characteristics			
AKI events	6533 (90%)	710 (10%)	7243
Number of patients	5151 (90%)	577 (10%)	5728
Median age, yr (IQR)	78 (67–86)	77 (69–84)	78 (67–85)
Males	2618 (51%)	297 (51%)	2915 (51%)
Comorbidities			
IHD	512 (10%)	51 (9%)	563 (10%)
Hypertension	77 (1%)	20 (3%)	97 (2%)
Heart failure	1262 (25%)	145 (25%)	1470 (26%)
Diabetes	1400 (27%)	184 (32%)	1584 (28%)
Liver disease	353 (7%)	59 (10%)	412 (7%)
Cirrhosis	117 (2%)	18 (3%)	135 (2%)
PVD	154 (3%)	17 (3%)	171 (3%)
Cancer	945 (18%)	113 (20%)	1058 (18%)
Renal stones	111 (2%)	9 (2%)	120 (2%)
*Chronic kidney disease*	623 (12%)	97 (17%)	720 (13%)
Stage 1	3 (0%)	1 (1%)	4 (1%)
Stage 2	10 (2%)	2 (2%)	12 (2%)
Stage 3	379 (61%)	57 (59%)	436 (61%)
Stage 4	132 (21%)	20 (21%)	152 (21%)
Stage 5	99 (16%)	17 (18%)	116 (16%)
Medications			
NSAIDs	687 (13%)	77 (13%)	764 (13%)
ACE inhibitors/ARBs	1756 (34%)	221 (38%)	1977 (35%)
*Diuretics*	2267 (44%)	271 (47%)	2531 (44%)
Thiazide	355 (16%)	55 (20%)	410 (16%)
Loop	1958 (86%)	229 (85%)	2187 (86%)
Potassium sparing	409 (18%)	48 (18%)	457 (18%)
Other	88 (4%)	7 (3%)	95 (4%)
Proton pump inhibitors	2831 (55%)	337 (58%)	3168 (55%)
Trimethoprim	205 (4%)	23 (4%)	228 (4%)
Aminoglycosides	886 (17%)	105 (18%)	991 (17%)
Vancomycin	278 (5%)	36 (6%)	314 (5%)
Penicillin	3256 (63%)	395 (68%)	3651 (64%)
First AKI stage			
Stage 1	4263 (83%)	265 (46%)	4528 (79%)
Stage 2	569 (11%)	174 (30%)	743 (13%)
Stage 3	319 (6%)	138 (24%)	457 (8%)
Status			
Died	1784 (35%)	210 (36%)	1994 (35%)

IQR: interquartile range; IHD: ischemic heart disease; PVD: peripheral vascular disease; NSAID: non-steroidal anti-inflammatory drug; ACE: angiotensin converting enzyme; ARB: angiotensin receptor blocker. Data presented as *n* (%), except for age, presented as median (IQR).

### Utilization of the AKI care plan (AKI-CP)

The AKI-CP was initiated in 9.8% of AKI events, with a greater proportion of comorbidities represented among AKI-CP initiated events, particularly diabetes, CKD and liver disease. Additionally, a higher proportion of AKI events with a higher first AKI stage was seen among AKI-CP initiated events (Table 1). A statistically significant increase in the utilization of the AKI-CP was noted across the study period (*p* < .05).

### All-cause inpatient mortality among AKI events

We used logistic regression to analyze several covariates identified *a priori* as potential confounders of the relationship between initiation of the AKI-CP and all-cause inpatient mortality: age, sex, comorbidities (including CKD), medications and first documented AKI stage. In the fully-adjusted multivariable analysis, initiation of the AKI-CP was associated with lower odds of all-cause inpatient mortality (odds ratio [OR]: 0.72; 95% confidence interval [CI] 0.57–0.91, Table 2) but not in the univariable analysis (OR: 0.91; 95% CI 0.74–1.13). Covariates independently associated with lower odds of inpatient mortality were a past medical history of renal stones (OR: 0.30; 95% CI 0.12–0.75), higher CKD stage, angiotensin converting enzyme (ACE) inhibitor or angiotensin receptor blocker (ARB) use (OR: 0.68; 95% CI 0.58–0.79), and aminoglycosides (OR: 0.55; 95% CI 0.45–0.68). AKI stages 2 and 3 were associated with higher odds of mortality compared to AKI stage 1 (OR: 1.98; 95% CI 1.65–2.37 and OR 1.66; 95% CI 1.31–2.09, respectively). Increasing age (OR: 1.04; 95% CI 1.03–1.04), heart failure, liver disease, cancer and penicillin and vancomycin use were also independently associated with increased odds of inpatient mortality (Table 2). In the fully-adjusted model, there was no evidence of multicollinearity.

**Table 2.** Univariable and multivariable adjusted analysis of covariates and all-cause inpatient mortality among AKI events.

	Univariable OR (95% CI)	Multivariable OR (95% CI)^a^
Age	1.04 (1.03, 1.04)	**1.04 (1.03, 1.04)**
Gender		
Female	1.00 (Reference)	1.00 (Reference)
Male	1.06 (0.93, 1.2)	1.02 (0.89, 1.17)
Comorbidities		
IHD	1.11 (0.9, 1.36)	1.02 (0.82, 1.27)
Hypertension	1.42 (0.95, 2.12)	1.19 (0.77, 1.83)
Heart failure	1.97 (1.73, 2.25)	**1.68 (1.42, 1.99)**
Diabetes	0.86 (0.75, 0.99)	0.93 (0.8, 1.07)
Liver disease	1.48 (1.19, 1.83)	**1.74 (1.3, 2.33)**
Cirrhosis	1.73 (1.26, 2.39)	1.46 (0.94, 2.27)
Peripheral vascular disease	1.27 (0.92, 1.75)	1.18 (0.84, 1.66)
Cancer	1.54 (1.33, 1.78)	**1.62 (1.39, 1.9)**
Renal stones	0.17 (0.07, 0.43)	**0.30 (0.12, 0.75)**
*Chronic kidney disease*	0.86 (0.72, 1.03)	**0.70 (0.57, 0.85)**
^b^Stage 1	–	–
Stage 2	0.72 (0.16, 3.16)	0.62 (0.13, 2.91)
Stage 3	0.96 (0.76, 1.21)	**0.75 (0.59, 0.96)**
Stage 4	0.9 (0.62, 1.29)	**0.65 (0.44, 0.95)**
Stage 5	0.65 (0.44, 0.96)	**0.61 (0.4, 0.93)**
Medications		
NSAIDs	0.77 (0.63, 0.94)	0.84 (0.68, 1.04)
ACE inhibitors/ARBs	0.72 (0.62, 0.83)	**0.68 (0.58, 0.79)**
*Diuretics*		
Thiazide	0.74 (0.57, 0.97)	0.76 (0.58, 1.01)
Loop	1.73 (1.53, 1.96)	1.27 (1.08, 1.49)
Potassium sparing	1.04 (0.83, 1.3)	**0.72 (0.56, 0.93)**
Other	1.55 (0.99, 2.41)	1.45 (0.91, 2.32)
Proton pump inhibitors	1.02 (0.9, 1.16)	0.95 (0.83, 1.08)
Trimethoprim	0.83 (0.59, 1.17)	0.75 (0.53, 1.06)
Aminoglycosides	0.61 (0.51, 0.74)	**0.55 (0.45, 0.68)**
Vancomycin	1.37 (1.08, 1.75)	**1.77 (1.37, 2.3)**
Penicillin	1.46 (1.28, 1.67)	**1.59 (1.38, 1.84)**
First AKI stage		
Stage 1	1.00 (Reference)	1.00 (Reference)
Stage 2	1.73 (1.46, 2.05)	**1.98 (1.65, 2.37)**
Stage 3	1.28 (1.03, 1.58)	**1.66 (1.31, 2.09)**
AKI care plan		
Not initiated	1.00 (Reference)	1.00 (Reference)
Initiated	0.91 (0.74, 1.13)	**0.72 (0.57, 0.91)**

OR: odds ratio; 95% CI: 95% confidence interval.

^a^Adjusted for age, comorbidities (IHD, hypertension, heart failure, diabetes, liver disease, cirrhosis, PVD, cancer, renal stones), medications (NSAIDs, ACE inhibitors/ARBs, diuretics [thiazide, loop, potassium sparing, other], proton pump inhibitors, trimethoprim, aminoglycosides, vancomycin, penicillin) and first documented AKI stage.

^b^Among AKI events with CKD stage 1 there were no inpatient deaths, hence these observations were excluded from the model.

Bold values indicate multivariable model *p* values < 0.05.

### Improvement in AKI stage, length of stay, survival post discharge, and impact of COVID-19

The median LOS among AKI events in the study was 9.0 days (IQR 4.3–17.2) and was longer among events where the AKI-CP was initiated (11.8 days, IQR 4.3–17.2) compared to those where it was not (8.8 d, IQR 4.1–17.2; Table 2). LOS was independently longer in the AKI-CP group even after adjusting for all covariates in the study, including inpatient mortality (*p* < .001, Supplementary Table 1). Increasing age, hypertension, diabetes, liver disease, peripheral vascular disease, and all medications analyzed (except for ACE inhibitors/ARBs and diuretics) were associated with a longer median LOS. Among AKI inpatients who died, heart failure, cancer and renal stones were associated with a shorter inpatient stay (*p* < .05, Supplementary Table 1).

Improvement in the AKI stage was defined as a reduction between the first and last AKI stage. Of the 7243 AKI events in the study, 775 had an improvement in the AKI stage: of these, 233 (3.2%) had an improvement by two stages and 542 (7.5%) by one stage (Table 3). Among AKI events with the AKI-CP initiated, 66 (9.3%) had an improvement by two AKI stages and 139 (19.6%) had an improvement in one AKI stage, compared to 167 (2.6%) and 403 (6.2%) events respectively for events that did not. Initiation of the AKI-CP was independently associated with improvement in the AKI stage (univariable OR: 4.25, 95% CI 3.53–5.10 versus multivariable OR: 4.27; 95% CI 3.54–5.14; Table 4).

**Table 3.** Progression of AKI stage and length of stay as per AKI-CP initiation.

	AKI-CP not initiated	AKI-CP initiated	Overall	*p*-value
Median length of stay, days (IQR)	8.8 (4.1–16.8)	11.8 (6.4–21.5)	9.0 (4.3–17.2)	*p* < .001^a^
Improvement in AKI stage				
Improvement by 2 stages	167 (2.6%)	66 (9.3%)	233 (3.2%)	*p* < .001^b^
Improvement by 1 stage	403 (6.2%)	139 (19.6%)	542 (7.5%)	
No difference or deterioration	5963 (91.3%)	505 (71.1%)	6468 (89.3%)	

IQR: interquartile range.

^a^Difference in medians calculated by Mann–Whitney *U* test.

^b^Difference in proportions calculated by chi-squared test.

**Table 4.** Univariable and multivariable adjusted analysis of covariates associated with change in AKI stage.

	Univariate OR (95% CI)	Multivariable OR (95% CI)^a^
Age	0.99 (0.99, 1)	0.99 (0.99, 1)
Gender		
Female	1.00 (Reference)	1.00 (Reference)
Male	1.16 (1, 1.35)	0.99 (0.95, 1.04)
Comorbidities		
Ischemic heart disease	0.81 (0.62, 1.07)	0.9 (0.68, 1.2)
Hypertension	1.2 (0.73, 1.97)	1.25 (0.73, 2.15)
Heart failure	0.74 (0.62, 0.89)	0.86 (0.69, 1.08)
Diabetes	1.01 (0.86, 1.19)	0.97 (0.82, 1.15)
Liver disease	1.29 (1, 1.67)	0.96 (0.68, 1.37)
Cirrhosis	1.53 (1.04, 2.25)	1.59 (0.93, 2.69)
Peripheral vascular disease	1.11 (0.74, 1.66)	1.17 (0.77, 1.78)
Cancer	1.26 (1.06, 1.51)	1.25 (1.03, 1.51)
Renal stones	1.23 (0.75, 1.99)	1.11 (0.67, 1.84)
*Chronic kidney disease*	0.79 (0.63, 0.99)	0.79 (0.62, 1)
Stage 1	2.69 (0.28, 25.94)	1.74 (0.17, 18.09)
Stage 2	0.54 (0.07, 4.09)	0.51 (0.07, 3.98)
Stage 3	0.83 (0.62, 1.11)	0.86 (0.63, 1.16)
Stage 4	0.68 (0.42, 1.11)	0.66 (0.4, 1.1)
Stage 5	0.81 (0.52, 1.25)	0.76 (0.48, 1.19)
Medications		
NSAIDs	0.96 (0.76, 1.2)	0.92 (0.73, 1.17)
ACE inhibitors/ARBs	1.1 (0.94, 1.29)	1.14 (0.96, 1.36)
*Diuretics*		
Thiazide	1.09 (0.82, 1.45)	1.02 (0.75, 1.38)
Loop	0.79 (0.68, 0.93)	0.91 (0.75, 1.11)
Potassium sparing	0.97 (0.74, 1.27)	1 (0.74, 1.37)
Other	0.55 (0.25, 1.18)	0.62 (0.28, 1.36)
Proton pump inhibitors	1.12 (0.96, 1.3)	1.09 (0.93, 1.28)
Trimethoprim	1.1 (0.77, 1.59)	1.22 (0.83, 1.78)
Aminoglycosides	1.02 (0.83, 1.24)	0.9 (0.72, 1.12)
Vancomycin	1.22 (0.91, 1.64)	1.14 (0.83, 1.55)
Penicillin	1.07 (0.92, 1.25)	1.07 (0.9, 1.26)
AKI care plan		
Not initiated	1.00 (Reference)	1.00 (Reference)
Initiated	4.25 (3.53, 5.1)	**4.27 (3.54, 5.14)**
Status		
Inpatient mortality	0.62 (0.49, 0.78)	**0.64 (0.5, 0.82)**

Bold values indicate multivariable model *p* values < 0.05.

**Figure 4.**
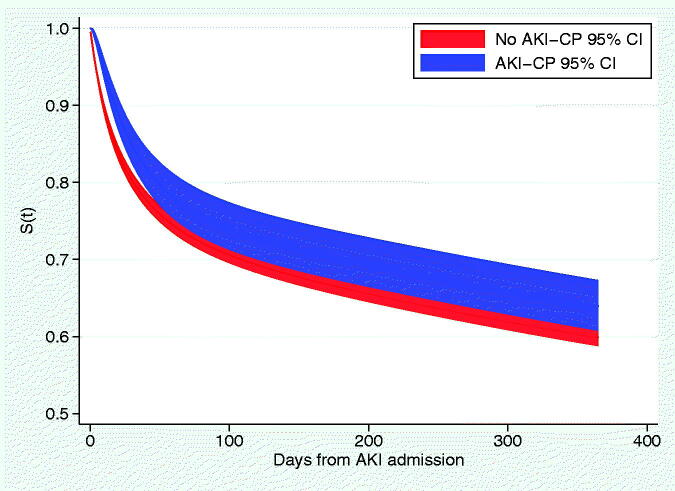
Standardized survival curves by initiation of the AKI-CP.

The crude survival analysis of initiation of the AKI-CP did not demonstrate an improvement in survival (unadjusted hazard ratio [HR]: 1.02; 95% CI 0.90–1.17). In the model, several covariates violated the proportional hazards assumption by significantly interacting with time (*p* < .05): age and sex; past medical history of hypertension, cancer, or CKD; use of thiazide, loop, or potassium sparing diuretics; proton pump inhibitor use; and aminoglycoside, vancomycin and trimethoprim medications. Consequently, Cox-proportional hazards were fitted with these variables as time-varying coefficients to calculate adjusted hazard ratios and RMSTs for use of the AKI-CP at different timepoints (Table 5). Median follow up was 251.9 d (IQR 57–590 d), and initiation of the AKI-CP was associated with a significantly lower hazard ratio of death at 30 days after the initial AKI event (HR 0.79; 95% CI 0.69–0.91), but not thereafter (Table 5), which was reflected in the standardized survival curves (Figure 4). The RMST was statistically significantly longer among those who had the AKI-CP initiated compared to those who did not (Table 5).

**Table 5.** Adjusted Cox-proportional hazard ratios and improvement in restricted mean survival time by completion of the AKI-CP at different timepoints.

Time after initial AKI event	Cox-proportional hazard ratio; HR (95% CI)^a^	Restricted mean survival time improvement with AKI-CP; days (95% CI)^a^
9 d i.e., median LOS	0.65 (0.54, 0.79)	0.31 (0.23, 0.38)
14 d	0.7 (0.59, 0.83)	0.56 (0.41, 0.72)
30 d	0.79 (0.69, 0.91)	1.35 (0.84, 1.86)
60 d	0.88 (0.77, 1.01)	2.55 (1.23, 3.87)
90 d	0.94 (0.82, 1.09)	3.64 (1.41, 5.87)
1 year	1.18 (0.96, 1.45)	14.49 (3.36, 25.62)

HR: hazard ratio; 95% CI: 95% confidence interval; LOS: length of stay.

^a^Adjusted for age, sex, IHD, heart failure, diabetes, liver disease, cirrhosis, PVD, cancer, renal stones, use of NSAIDs, ACE/ARBs, diuretics (thiazide, loop, potassium sparing and other), proton pump inhibitors, antibiotics (trimethoprim, aminoglycosides, vancomycin and penicillin). Age, sex, hypertension, cancer, CKD, thiazides, loop diuretics, potassium sparing diuretics, proton pump inhibitors, aminoglycosides, vancomycin, trimethoprim, first AKI stage and initiation of the AKI-CP were treated as time-varying coefficients.

Within the complete case analysis study cohort, there were 2750 AKI events with an RT-PCR test for SARS-CoV-2 among 2025 patients, who were 51% male with a median age of 78 (67–85) years. 130/2025 patients (6%) were positive (Supplementary Table 2) for SARS-CoV-2, and among AKI events, a positive test was independently associated with increased inpatient mortality (univariable OR: 5.47, 95% CI 3.59–7.59 versus multivariable OR: 5.54, 95% CI 3.91–7.89, Supplementary Table 3), improvement in AKI stage (univariable OR: 1.79, 95% CI 1.18–2.70 versus multivariable OR: 1.82, 95% CI 1.18–2.80, Supplementary Table 4), and longer LOS (univariable RR: 1.49, 95% CI 1.28–1.72 versus multivariable OR: 1.61, 95% CI 1.41–1.84, Supplementary Table 5). Limiting the analysis to the first AKI event for which a patient had an RT-PCR test for SARS-CoV-2, testing positive for COVID-19 remained independently associated with increased inpatient mortality and LOS, but not improvement in the AKI stage. AKI-CP utilization remained associated with an improvement in inpatient mortality and was independently associated with an improvement in AKI stage but a longer LOS among AKI patients tested for SARS-CoV-2 (Supplementary Tables 3–5). Among AKI events with a positive SARS-CoV-2 test, AKI-CP utilization remained associated with an improvement in AKI stage in the univariable (univariable OR: 5.05, 95% CI 2.00–12.74) but not the multivariable model (multivariable OR: 2.05, 95% CI 0.08–52.54) and did not have mortality benefit (univariable OR: 0.43, 95% CI 0.17–1.09; multivariable OR: 0.51, 95% CI 0.16–1.67).

### Audit of awareness and subjective utilization of the AKI order set

The initial survey of awareness and subjective utilization of the AKI order set among junior doctors in October 2020 had 53 respondents (Supplementary Table 6). 66% of respondents were aware of the AKI order set. Among these, on a Likert scale of self-reported frequency of order set utilization from 1 (Never) to 5 (Always), the median score was 3. Other than lack of awareness, the most commonly reported barriers to utilization of the order set were the belief that it was unnecessary as interventions can be ordered separately (32%), lack of senior awareness and encouragement (28%), and electronic alert fatigue (28%). The most commonly suggested interventions to improve uptake were formal educational sessions (66%), increased senior awareness (60%) and posters (49%). After a campaign of interventions including posters and educational sessions targeted at both senior and junior doctors, the survey was repeated. At this time, 100% of respondents were aware of the AKI order set; the median Likert score for self-reported frequency of order set utilization was 3. The difference in self-reported order set utilization between study respondents was found to be non-significant, using a two-tailed Mann-Whitney *U* test (*z* = −1.236, *p* = .2136). There was no significant increase in the use of the AKI order set (11.1%; 95% CI 9.81–12.6%) compared to the 12 months prior (9.9%; 95% CI 8.79–11.1%; *p* = .172, chi-squared).

## Discussion

This study demonstrates that the use of an evidence-based, integrated electronic detection/alerting system associated with lower inpatient all-cause mortality, improvement in AKI stage and lower hazard for death up to 30 days post initial AKI event in a real-world secondary care setting consisting of a predominantly elderly population, who are at increased risk of adverse outcomes following AKI [7]. These benefits came at the cost of longer LOS, although it is possible that AKI patients who stayed longer in the hospital had more opportunities for the AKI order set to be requested or represented a more medically complicated cohort. With the exception of longer LOS and low utilization of the AKI order set, our findings reinforce the limited existing evidence of the effectiveness of electronic alerting and care bundle ordering on AKI outcome measures (renal function and mortality) [12,14,19,23], and strengthen the case for implementation of such interventions in real-world settings. In particular, we investigated an electronic alert directly linked to the ordering of a recommended care bundle, and our study reinforces the finding of Haase et al. that electronic alerting coupled with treatment recommendations is most likely to be effective [18], though of course direct comparison with an isolated electronic alert not coupled with treatment recommendations was not possible within our study design.

Several comorbidities and medications were associated with all-cause inpatient mortality in the univariable but not the multivariable analyses and *vice versa*, likely representing the impact of confounding by other factors (Table 2). For example, although the finding that a history of NSAID use and thiazide diuretics were each associated with lower odds of mortality in the univariable mode was unexpected given their associations with AKI [24,25], this association was not apparent in the multivariable model. Similarly, the crude statistically significant association between diabetes and all-cause inpatient mortality was not seen in the multivariable model, which is in keeping with conflicting data in the literature on this association [26,27]. By contrast, advancing CKD stage was associated with reduced all-cause inpatient mortality in the multivariable but not in the univariable model in our study, which is similar to a retrospective study of 82,711 hospitalized AKI events from the United States Department of Veterans Affairs healthcare system by Lafrance and Miller. that found that although AKI was independently associated with all-cause mortality risk, this diminished with advancing CKD stage [28]. Other studies of cohorts of similar patients with AKI have found the opposite association [29]. Further studies investigating the impact of CKD on AKI outcomes among inpatients are likely required.

Utilization of the AKI order set remained persistently low throughout the period studied. Disappointingly, there was no statistically significant improvement in subjective or objective utilization after a campaign of interventions, despite 100% awareness. Possible explanations for this may include the rapid turnover of junior doctors and the high cognitive burden of multiple alerts and care bundles, and this is in keeping with previous work investigating the obstacles to the implementation of electronic AKI systems in resource-pressured clinical settings such as NHS hospitals [30]. While we have demonstrated significant positive impacts of care bundle ordering, this is unlikely to translate to improvement in real-world patient outcomes unless the challenge of utilization in clinical practice can be met.

Strengths of this study include a large dataset encompassing all AKI episodes at a UK hospital over three years, collected through direct interrogation of the hospital database. The findings are therefore likely to be robust and representative of real-world secondary center practice. By comparing patients in whom the order set was requested to those in whom it was not across a single time period, we avoid potential confounding factors such as broader changes in organizational policy, which commonly affect before-and-after studies of AKI alerting and care bundle systems.

The study does however have several limitations. The dataset is taken from a single hospital (a secondary center in a relatively affluent area with a largely older Caucasian population) and may not be generalizable to tertiary centers or those serving populations with different demographics. Moreover, although we adjusted for several confounding variables for the association between the use of the AKI order set and the outcomes of interest, our findings could still be influenced by residual unmeasured confounders.

Following the introduction of the AKI order set, further work has been undertaken by introducing a Clinical Decision Support system. For patients in whom AKI is detected, the electronic system now prompts clinicians to open an interface which recommends patient-specific interventions based on answers to simple questions. We hope that future research will demonstrate further improvement in both process and outcome measures by tailoring care to each patient.

## Supplementary Material

Supplemental Material

## Data Availability

The data underlying this article cannot be shared publicly due to patient confidentiality requirements as unique patient identifiers were used to ascertain outcomes.

## References

[1] Lameire NH, Bagga A, Cruz D, et al. Acute kidney injury: an increasing global concern. Lancet. 2013;382(9887):1–9. doi: 10.1016/s0140-6736(13)60647-9. 23727171

[2] Hoste EAJ, Kellum JA, Selby NM, et al. Global epidemiology and outcomes of acute kidney injury. Nat Rev Nephrol. 2018;14(10):607–625. doi: 10.1038/s41581-018-0052-0. 30135570

[3] NICE. Acute kidney injury: quality standard [QS76]. 2014.

[4] Challiner R, Ritchie JP, Fullwood C, et al. Incidence and consequence of acute kidney injury in unselected emergency admissions to a large acute UK hospital trust. BMC Nephrol. 2014;15(1):84. doi: 10.1186/1471-2369-15-84. 24885247 PMC4046061

[5] Nadim MK, Forni LG, Mehta RL, et al. COVID-19-associated acute kidney injury: consensus report of the 25th acute disease quality initiative (ADQI) workgroup. Nat Rev Nephrol. 2020;16(12):747–764. doi: 10.1038/s41581-020-00356-5. 33060844 PMC7561246

[6] Bucaloiu ID, Kirchner HL, Norfolk ER, et al. Increased risk of death and de novo chronic kidney disease following reversible acute kidney injury. Kidney Int. 2012;81(5):477–485. doi: 10.1038/ki.2011.405. 22157656

[7] Coca SG, Singanamala S, Parikh CR. Chronic kidney disease after acute kidney injury: a systematic review and meta-analysis. Kidney Int. 2012;81(5):442–448. doi: 10.1038/ki.2011.379. 22113526 PMC3788581

[8] National Confidential Enquiry into Patient Outcome and Death (NCEPOD). Acute kidney injury: adding insult to injury. London: National Confidential Enquiry into Patient Outcome and Death (NCEPOD); 2009.

[9] Selby NM, Crowley L, Fluck RJ, et al. Use of electronic results reporting to diagnose and monitor AKI in hospitalized patients. Clin J Am Soc Nephrol. 2012;7(4):533–540. doi: 10.2215/cjn.08970911. 22362062

[10] Selby NM. Electronic alerts for acute kidney injury. Curr Opin Nephrol Hypertens. 2013;22(6):637–642. doi: 10.1097/MNH.0b013e328365ae84. 24100217

[11] Porter CJ, Juurlink I, Bisset LH, et al. A real-time electronic alert to improve detection of acute kidney injury in a large teaching hospital. Nephrol Dial Transplant. 2014;29(10):1888–1893. doi: 10.1093/ndt/gfu082. 24744280

[12] Kolhe NV, Staples D, Reilly T, et al. Impact of compliance with a care bundle on acute kidney injury outcomes: a prospective observational study. PLOS One. 2015;10(7):e0132279. doi: 10.1371/journal.pone.0132279. 26161979 PMC4498890

[13] Lachance P, Villeneuve PM, Rewa OG, et al. Association between e-alert implementation for detection of acute kidney injury and outcomes: a systematic review. Nephrol Dial Transplant. 2017;32(2):265–272. doi: 10.1093/ndt/gfw424. 28088774 PMC6251638

[14] Selby NM, Casula A, Lamming L, et al. An Organizational-Level program of intervention for AKI: a pragmatic stepped wedge cluster randomized trial. J Am Soc Nephrol. 2019;30(3):505–515. doi: 10.1681/asn.2018090886. 31058607 PMC6405151

[15] Wilson FP, Martin M, Yamamoto Y, et al. Electronic health record alerts for acute kidney injury: multicenter, randomized clinical trial. BMJ. 2021;372:m4786. doi: 10.1136/bmj.m4786. 33461986 PMC8034420

[16] Holmes J, Donovan K, Geen J, et al. Acute kidney injury demographics and outcomes: changes following introduction of electronic acute kidney injury alerts-an analysis of a national dataset. Nephrol Dial Transplant. 2021;36(8):1433–1439. doi: 10.1093/ndt/gfaa071. 32514532

[17] Schaubroeck HAI, Vargas D, Vandenberghe W, et al. Impact of AKI care bundles on kidney and patient outcomes in hospitalized patients: a systematic review and meta-analysis. BMC Nephrol. 2021;22(1):335. doi: 10.1186/s12882-021-02534-4. 34625046 PMC8501614

[18] Haase M, Kribben A, Zidek W, et al. Electronic alerts for acute kidney injury. Dtsch Arztebl Int. 2017;114(1-02):1–8. doi: 10.3238/arztebl.2017.0001. 28143633 PMC5399999

[19] Al-Jaghbeer M, Dealmeida D, Bilderback A, et al. Clinical decision support for in-hospital AKI. J Am Soc Nephrol. 2018;29(2):654–660. doi: 10.1681/asn.2017070765. 29097621 PMC5791078

[20] Summary of recommendation statements. Kidney Int Suppl. 2012;2(1):8–12. doi: 10.1038/kisup.2012.7. PMC4089654 25018916

[21] Lambert PC, Royston P. Further development of flexible parametric models for survival analysis. Stata J. 2009;9(2):265–290. doi: 10.1177/1536867X0900900206.

[22] Royston P, Parmar MK. Flexible parametric proportional-hazards and proportional-odds models for censored survival data, with application to prognostic modelling and estimation of treatment effects. Stat Med. 2002;21(15):2175–2197. doi: 10.1002/sim.1203. 12210632

[23] Kolhe NV, Reilly T, Leung J, et al. A simple care bundle for use in acute kidney injury: a propensity score-matched cohort study. Nephrol Dial Transplant. 2016;31(11):1846–1854. doi: 10.1093/ndt/gfw087. 27190331

[24] Ungprasert P, Cheungpasitporn W, Crowson CS, et al. Individual non-steroidal anti-inflammatory drugs and risk of acute kidney injury: a systematic review and meta-analysis of observational studies. Eur J Intern Med. 2015;26(4):285–291. doi: 10.1016/j.ejim.2015.03.008. 25862494

[25] Mehta RL, Pascual MT, Soroko S, et al. Diuretics, mortality, and nonrecovery of renal function in acute renal failure. JAMA. 2002;288(20):2547–2553. doi: 10.1001/jama.288.20.2547. 12444861

[26] Johnson F, Phillips D, Talabani B, et al. The impact of acute kidney injury in diabetes mellitus. Nephrology. 2016;21(6):506–511. doi: 10.1111/nep.12649. 26452246

[27] Tan L, Chen L, Jia Y, et al. Impact of diabetes mellitus on short-term prognosis, length of stay, and costs in patients with acute kidney injury: a nationwide survey in China. PLOS One. 2021;16(5):e0250934. doi: 10.1371/journal.pone.0250934. 33939742 PMC8092800

[28] Lafrance JP, Miller DR. Acute kidney injury associates with increased long-term mortality. J Am Soc Nephrol. 2010;21(2):345–352. doi: 10.1681/ASN.2009060636. 20019168 PMC2834549

[29] Sawhney S, Mitchell M, Marks A, et al. Long-term prognosis after acute kidney injury (AKI): what is the role of baseline kidney function and recovery? A systematic review. BMJ Open. 2015;5(1):e006497–e006497. doi: 10.1136/bmjopen-2014-006497. PMC4289733 25564144

[30] Bailey S, Hunt C, Brisley A, et al. Implementation of clinical decision support to manage acute kidney injury in secondary care: an ethnographic study. BMJ Qual Saf. 2020;29(5):382–389. doi: 10.1136/bmjqs-2019-009932. PMC7241968 31796574

