# Supplementary material for: Impact of electronic AKI alert/care bundle on AKI inpatient outcomes: a retrospective single-center cohort study
Source: Ren Fail. 2024 Feb 12;46(1):2313177. doi: 10.1080/0886022X.2024.2313177 (PMC10863540; doi:10.1080/0886022X.2024.2313177)
Supplement: Supplemental Material [file IRNF_A_2313177_SM5567.pdf]

Supplemental Material

Supplemental Table 1. Factors associated with length of stay among AKI events by truncated negative binomial regression

Supplemental Table 2. Factors associated with improvement in AKI stage

Supplemental Table 3. Baseline characteristics of patients with a SARS-CoV-2 PCR test by AKI-CP initiation status

Supplemental Table 4. Univariate and multivariable adjusted analysis of covariates and all-cause inpatient mortality among AKI events with a SARS-CoV-2 PCR test

Supplemental Table 5. Univariate and multivariable adjusted analysis of covariates and improvement in AKI stage among AKI events with a SARS-CoV-2 PCR test

Supplemental Table 6. Factors associated with length of stay among AKI events with a SARS-CoV-2 PCR test by truncated negative binomial regression

Supplemental Table 7. Characteristics of the respondents to the survey of AKI-CP initiation

Supplemental Table 1. Factors associated with length of stay among AKI events by truncated negative binomial regression

	Univariate RR (95% CI)	Multivariable RR (95% CI)[a]
**Age**	1.01 (1.01, 1.01)	1.01 (1.01, 1.01)
**Gender**		
Female	1.00 (Reference)	1.00 (Reference)
Male	1.02 (0.97, 1.07)	0.99 (0.95, 1.04)
**Comorbidities**		
Ischaemic heart disease	1.16 (1.07, 1.27)	1.07 (0.99, 1.16)
Hypertension	1.36 (1.13, 1.63)	1.18 (1, 1.39)
Heart failure	1.13 (1.07, 1.2)	0.92 (0.87, 0.98)
Diabetes	1.09 (1.03, 1.15)	1.06 (1, 1.11)
Liver disease	1.18 (1.07, 1.3)	1.13 (1.02, 1.26)
Cirrhosis	1.14 (0.98, 1.33)	0.95 (0.8, 1.12)
Peripheral vascular disease	1.25 (1.09, 1.45)	1.15 (1.02, 1.31)
Cancer	0.94 (0.88, 1)	0.93 (0.88, 0.98)
Renal stones	0.76 (0.63, 0.91)	0.67 (0.57, 0.79)
Chronic kidney disease	1.04 (0.97, 1.12)	0.93 (0.87, 1)
Stage 1	0.96 (0.32, 2.88)	0.64 (0.24, 1.66)
Stage 2	1.17 (0.67, 2.02)	1.09 (0.68, 1.76)
Stage 3	1.13 (1.02, 1.24)	0.98 (0.9, 1.07)
Stage 4	1 (0.87, 1.16)	0.9 (0.79, 1.03)
Stage 5	0.88 (0.76, 1.01)	0.84 (0.74, 0.96)

**Medications**		
NSAIDs	1.18 (1.09, 1.28)	1.1 (1.03, 1.18)
ACE inhibitors/ARBs	1.05 (0.99, 1.11)	0.96 (0.91, 1.01)
Diuretics		
Thiazide	1.3 (1.17, 1.44)	1.25 (1.14, 1.37)
Loop	1.36 (1.29, 1.43)	1.25 (1.18, 1.32)
Potassium sparing	1.33 (1.21, 1.46)	1.2 (1.1, 1.31)
Other	1.19 (0.97, 1.47)	1.18 (0.98, 1.41)
Proton pump inhibitors	1.38 (1.31, 1.46)	1.28 (1.22, 1.34)
Trimethoprim	1.11 (0.97, 1.26)	1.12 (1, 1.26)
Aminoglycosides	1.54 (1.44, 1.65)	1.25 (1.17, 1.33)
Vancomycin	2.6 (2.34, 2.88)	2.56 (2.33, 2.82)
Penicillin	1.7 (1.62, 1.79)	1.52 (1.45, 1.6)
**First AKI stage**		
Stage 1	1.00 (Reference)	1.00 (Reference)
Stage 2	0.93 (0.86, 1.01)	0.95 (0.89, 1.02)
Stage 3	0.89 (0.82, 0.98)	0.96 (0.88, 1.04)
**AKI care plan**		
Not initiated	1.00 (Reference)	1.00 (Reference)
Initiated	1.36 (1.25, 1.48)	1.27 (1.18, 1.37)
**Status**		
Inpatient mortality	0.96 (0.9, 1.03)	0.87 (0.81, 0.92)

[a]Adjusted for age, comorbidities (IHD, hypertension, heart failure, diabetes, liver disease, cirrhosis, PVD, cancer, renal stones, CKD), medications (NSAIDs, ACE inhibitors/ARBs, diuretics [thiazide, loop, potassium

sparing, other], proton pump inhibitors, trimethoprim, aminoglycosides, vancomycin, penicillin), first documented AKI stage, AKI-CP and inpatient mortality. RR, rate ratio; NSAID, non-steroidal anti-inflammatory drug; ACE, angiotensin converting enzyme; ARB, angiotensin receptor blocker.

Supplemental Table 2. Factors associated with improvement in AKI stage

	Univariate OR (95% CI)	Multivariable OR (95% CI)[a]
**Age**	0.99 (0.99, 1)	0.99 (0.99, 1)
**Gender**		
Female	1.00 (Reference)	1.00 (Reference)
Male	1.16 (1, 1.35)	0.99 (0.95, 1.04)
**Comorbidities**		
Ischaemic heart disease	0.81 (0.62, 1.07)	0.9 (0.68, 1.2)
Hypertension	1.2 (0.73, 1.97)	1.25 (0.73, 2.15)
Heart failure	0.74 (0.62, 0.89)	0.86 (0.69, 1.08)
Diabetes	1.01 (0.86, 1.19)	0.97 (0.82, 1.15)
Liver disease	1.29 (1, 1.67)	0.96 (0.68, 1.37)
Cirrhosis	1.53 (1.04, 2.25)	1.59 (0.93, 2.69)
Peripheral vascular disease	1.11 (0.74, 1.66)	1.17 (0.77, 1.78)
Cancer	1.26 (1.06, 1.51)	1.25 (1.03, 1.51)
Renal stones	1.23 (0.75, 1.99)	1.11 (0.67, 1.84)
Chronic kidney disease	0.79 (0.63, 0.99)	0.79 (0.62, 1)
Stage 1	2.69 (0.28, 25.94)	1.74 (0.17, 18.09)
Stage 2	0.54 (0.07, 4.09)	0.51 (0.07, 3.98)
Stage 3	0.83 (0.62, 1.11)	0.86 (0.63, 1.16)
Stage 4	0.68 (0.42, 1.11)	0.66 (0.4, 1.1)
Stage 5	0.81 (0.52, 1.25)	0.76 (0.48, 1.19)

**Medications**		
NSAIDs	0.96 (0.76, 1.2)	0.92 (0.73, 1.17)
ACE inhibitors/ARBs	1.1 (0.94, 1.29)	1.14 (0.96, 1.36)
Diuretics		
Thiazide	1.09 (0.82, 1.45)	1.02 (0.75, 1.38)
Loop	0.79 (0.68, 0.93)	0.91 (0.75, 1.11)
Potassium sparing	0.97 (0.74, 1.27)	1 (0.74, 1.37)
Other	0.55 (0.25, 1.18)	0.62 (0.28, 1.36)
Proton pump inhibitors	1.12 (0.96, 1.3)	1.09 (0.93, 1.28)
Trimethoprim	1.1 (0.77, 1.59)	1.22 (0.83, 1.78)
Aminoglycosides	1.02 (0.83, 1.24)	0.9 (0.72, 1.12)
Vancomycin	1.22 (0.91, 1.64)	1.14 (0.83, 1.55)
Penicillin	1.07 (0.92, 1.25)	1.07 (0.9, 1.26)
**AKI care plan**		
Not initiated	1.00 (Reference)	1.00 (Reference)
Initiated	4.25 (3.53, 5.1)	**4.27 (3.54, 5.14)**
**Status**		
Inpatient mortality	0.62 (0.49, 0.78)	**0.64 (0.5, 0.82)**

[a]Adjusted for age, comorbidities (IHD, hypertension, heart failure, diabetes, liver disease, cirrhosis, PVD, cancer, renal stones, CKD), medications (NSAIDs, ACE inhibitors/ARBs, diuretics [thiazide, loop, potassium sparing, other], proton pump inhibitors, trimethoprim, aminoglycosides, vancomycin, penicillin), first documented AKI stage, and inpatient mortality. OR, odds ratio; NSAID, non-steroidal anti-inflammatory drug; ACE, angiotensin converting enzyme; ARB, angiotensin receptor blocker.

Supplemental Table 3. Baseline characteristics of patients with a SARS-CoV-2 PCR test by AKI-CP initiation status

	AKI-CP not initiated	AKI-CP initiated	Total
**Characteristics**			
**No. of events**	2431 (88%)	319 (12%)	2750 (100%)
**No. of patients**	1782 (88%)	243 (12%)	2025 (100%)
**Men, %**	924 (51%)	117 (51%)	1041 (51%)
**Median age, years (IQR)**	78 (67 to 86)	77 (69 to 84)	78 (67 to 85)
**Age group, yr, %**			
18-59	282 (16%)	29 (12%)	311 (15%)
60-69	220 (12%)	28 (12%)	248 (12%)
70-79	475 (27%)	67 (28%)	542 (27%)
80-89	557 (31%)	86 (35%)	643 (32%)
90+	248 (14%)	33 (14%)	281 (14%)
**Comorbidities**			
IHD	190 (11%)	17 (7%)	207 (10%)
Hypertension	39 (2%)	8 (3%)	47 (2%)
Heart failure	488 (27%)	68 (28%)	556 (27%)
Diabetes	520 (29%)	81 (33%)	601 (30%)
Liver disease	134 (8%)	24 (10%)	158 (8%)
Cirrhosis	42 (2%)	6 (2%)	48 (2%)
PVD	47 (3%)	3 (1%)	50 (2%)
Cancer	300 (17%)	53 (22%)	353 (17%)

Chronic kidney disease	259 (15%)	48 (20%)	307 (15%)
Stage 1	2 (1%)	0 (0%)	4 (1%)
Stage 2	6 (2%)	2 (4%)	12 (4%)
Stage 3	171 (66%)	29 (60%)	436 (142%)
Stage 4	55 (21%)	9 (19%)	152 (50%)
Stage 5	25 (10%)	8 (17%)	116 (38%)
Renal stones	42 (2%)	3 (1%)	45 (2%)
**Medications**			
NSAIDs	242 (14%)	37 (15%)	279 (14%)
ACE inhibitors/ARBs	655 (37%)	95 (39%)	750 (37%)
Diuretics	847 (48%)	120 (49%)	967 (48%)
Thiazide	141 (17%)	24 (20%)	165 (17%)
Loop	743 (88%)	102 (85%)	845 (87%)
Potassium sparing	161 (19%)	19 (16%)	180 (19%)
Other	26 (3%)	4 (3%)	30 (3%)
Proton pump inhibitors	1086 (61%)	144 (59%)	1230 (61%)
Trimethoprim	78 (4%)	5 (2%)	83 (4%)
Aminoglycosides	297 (17%)	43 (18%)	340 (17%)
Vancomycin	126 (7%)	15 (6%)	141 (7%)
Penicillin	1132 (64%)	172 (71%)	1304 (64%)
**First AKI stage**			
Stage 1	1481 (83%)	122 (50%)	1603 (79%)
Stage 2	193 (11%)	61 (25%)	254 (13%)

Stage 3	108 (6%)	60 (25%)	168 (8%)
**Status**			
Died	540 (30%)	83 (34%)	623 (31%)
COVID-19 PCR positive	106 (6%)	24 (10%)	130 (6%)

IQR, interquartile range; IHD, ischaemic heart disease; PVD, peripheral vascular disease; NSAID, non-steroidal anti-inflammatory drug; ACE, angiotensin converting enzyme; ARB, angiotensin receptor blocker. Data presented as *n* (%), except for age, presented as median (IQR)

Supplemental Table 4. Univariate and multivariable adjusted analysis of covariates and all-cause inpatient mortality among AKI events with a SARS-CoV-2 PCR test

	**Univariate OR (95% CI)**	**Multivariable OR (95% CI)**[a]
**Age**	1.03 (1.02, 1.04)	1.03 (1.02, 1.04)
**Gender**		
Female	1.00 (Reference)	1.00 (Reference)
Male	1.14 (0.92, 1.4)	1.03 (0.82, 1.3)
**Comorbidities**		
Ischaemic heart disease	1.59 (1.16, 2.17)	1.52 (1.08, 2.14)
Hypertension	1.59 (1.16, 2.17)	1.45 (0.78, 2.68)
Heart failure	1.59 (1.28, 1.99)	1.46 (1.09, 1.96)
Diabetes	0.92 (0.73, 1.16)	0.97 (0.76, 1.25)
Liver disease	1.36 (0.96, 1.92)	1.68 (1.05, 2.69)
Cirrhosis	1.47 (0.85, 2.52)	1.22 (0.58, 2.56)
Peripheral vascular disease	1.74 (1.02, 2.98)	1.62 (0.9, 2.91)
Cancer	1.85 (1.46, 2.36)	1.94 (1.48, 2.54)
Renal stones	0.2 (0.05, 0.8)	0.23 (0.05, 1.02)
Chronic kidney disease	0.89 (0.67, 1.18)	0.72 (0.53, 0.99)
Stage 1[b]	0 (0, 0)	0 (0, 0)
Stage 2[b]	0 (0, 0)	0 (0, 0)
Stage 3	1 (0.71, 1.42)	0.81 (0.56, 1.18)
Stage 4	0.72 (0.39, 1.32)	0.51 (0.26, 1)
Stage 5	0.89 (0.49, 1.61)	0.78 (0.4, 1.52)

**Medications**		
NSAID	1.07 (0.78, 1.45)	1.26 (0.91, 1.75)
ACE inhibitors/ARBs	0.72 (0.57, 0.91)	0.69 (0.53, 0.89)
Diuretics	0 (0, 0)	
Thiazide	0.9 (0.59, 1.37)	0.95 (0.6, 1.49)
Loop	1.49 (1.21, 1.85)	1.17 (0.89, 1.54)
Potassium sparing	1.09 (0.77, 1.55)	0.92 (0.61, 1.38)
Other	1.88 (0.88, 4.02)	1.9 (0.85, 4.24)
Proton pump inhibitors	1.13 (0.9, 1.4)	1.1 (0.87, 1.39)
Trimethoprim	0.99 (0.59, 1.68)	0.98 (0.56, 1.7)
Aminoglycosides	0.83 (0.61, 1.12)	0.72 (0.51, 1.01)
Vancomycin	1.61 (1.13, 2.29)	1.97 (1.34, 2.91)
Penicillin	1.38 (1.1, 1.72)	1.42 (1.1, 1.82)
**First AKI stage**		
Stage 1	1.00 (Reference)	1.00 (Reference)
Stage 2	1.77 (1.33, 2.35)	1.84 (1.34, 2.52)
Stage 3	1.52 (1.08, 2.14)	1.94 (1.32, 2.86)
**AKI care plan**		
Not initiated	1.00 (Reference)	1.00 (Reference)
Initiated	0.89 (0.63, 1.25)	0.65 (0.44, 0.95)
**Status**		
COVID-19 status	5.47 (3.95, 7.59)	5.54 (3.91, 7.86)

[a] Adjusted for age, comorbidities (IHD, hypertension, heart failure, diabetes, liver disease, cirrhosis, PVD, cancer, renal stones, CKD), medications (NSAIDs, ACE inhibitors/ARBs, diuretics [thiazide, loop, potassium

sparing, other], proton pump inhibitors, trimethoprim, aminoglycosides, vancomycin, penicillin), first documented AKI stage and COVID-19 status.

[b] Among AKI events with CKD stages 1 and 2 there were no inpatient deaths, hence these observations were excluded from the model.

Supplemental Table 5. Univariate and multivariable adjusted analysis of covariates and improvement in AKI stage among AKI events with a SARS-CoV-2 PCR test

	**Univariate OR (95% CI)**	**Multivariable OR (95% CI)[a]**
**Age**	0.99 (0.98, 1)	0.99 (0.98, 1)
**Gender**		
Female	1.00 (Reference)	1.00 (Reference)
Male	0.96 (0.76, 1.22)	0.96 (0.75, 1.22)
**Comorbidities**		
Ischaemic heart disease	0.86 (0.57, 1.3)	0.99 (0.65, 1.53)
Hypertension	1.11 (0.57, 2.18)	1.26 (0.6, 2.63)
Heart failure	0.82 (0.63, 1.07)	1.01 (0.71, 1.44)
Diabetes	0.95 (0.73, 1.22)	0.95 (0.73, 1.25)
Liver disease	1.14 (0.76, 1.7)	0.9 (0.53, 1.53)
Cirrhosis	1.29 (0.69, 2.4)	1.38 (0.59, 3.21)
Peripheral vascular disease	0.63 (0.27, 1.46)	0.74 (0.31, 1.77)
Cancer	1.3 (0.98, 1.73)	1.33 (0.98, 1.8)
Renal stones	1.77 (0.91, 3.44)	1.73 (0.86, 3.48)
Chronic kidney disease	0.81 (0.58, 1.11)	0.87 (0.62, 1.23)
Stage 1	7.48 (0.47, 119.96)	8.17 (0.49, 136.82)
Stage 2[b]	1 (0, 0)	1 (0, 0)
Stage 3	1 (0.68, 1.47)	1.14 (0.76, 1.71)
Stage 4	0.69 (0.34, 1.38)	0.7 (0.34, 1.44)
Stage 5	0.41 (0.17, 1.02)	0.41 (0.16, 1.03)

**Medications**		
NSAID	1.08 (0.77, 1.52)	1.03 (0.72, 1.47)
ACE inhibitors/ARBs	1.06 (0.83, 1.37)	1.1 (0.84, 1.44)
Diuretics	0 (0, 0)	
Thiazide	1.3 (0.85, 1.97)	1.23 (0.78, 1.93)
Loop	0.75 (0.58, 0.95)	0.76 (0.56, 1.04)
Potassium sparing	1.06 (0.71, 1.57)	1.18 (0.74, 1.87)
Other	0.44 (0.11, 1.84)	0.45 (0.1, 1.95)
Proton pump inhibitors	0.97 (0.76, 1.24)	1.02 (0.79, 1.31)
Trimethoprim	1.35 (0.8, 2.29)	1.59 (0.92, 2.75)
Aminoglycosides	0.97 (0.7, 1.34)	0.98 (0.68, 1.42)
Vancomycin	0.86 (0.54, 1.37)	0.82 (0.5, 1.34)
Penicillin	0.88 (0.69, 1.12)	0.83 (0.64, 1.08)
**AKI care plan**		
Not initiated	1.00 (Reference)	1.00 (Reference)
Initiated	3.72 (2.81, 4.92)	3.92 (2.94, 5.24)
**Status**		
COVID-19 status	1.79 (1.18, 2.7)	2.02 (1.29, 3.16)
Inpatient mortality	0.7 (0.48, 1.01)	0.68 (0.46, 1.01)

[a] Adjusted for age, comorbidities (IHD, hypertension, heart failure, diabetes, liver disease, cirrhosis, PVD, cancer, renal stones, CKD), medications (NSAIDs, ACE inhibitors/ARBs, diuretics [thiazide, loop, potassium sparing, other], proton pump inhibitors, trimethoprim, aminoglycosides, vancomycin, penicillin), first documented AKI stage, COVID-19 status and inpatient mortality.

[b] Among AKI events with CKD stage 2 there were no inpatient deaths, hence these observations were excluded from the model.

Supplemental Table 6. Factors associated with length of stay among AKI events with a SARS-CoV-2 PCR test by truncated negative binomial regression

	**Univariate RR (95% CI)**	**Multivariable RR (95% CI)[a]**
**Age**	1 (1, 1.01)	1.01 (1, 1.01)
**Gender**		
Female	1.00 (Reference)	1.00 (Reference)
Male	1 (0.93, 1.08)	0.99 (0.93, 1.05)
**Comorbidities**		
Ischaemic heart disease	1.17 (1.04, 1.31)	1.1 (0.99, 1.22)
Hypertension	1.33 (1.08, 1.64)	1.19 (0.98, 1.44)
Heart failure	1.07 (0.99, 1.16)	0.89 (0.82, 0.97)
Diabetes	1.02 (0.94, 1.1)	1 (0.93, 1.07)
Liver disease	1.17 (1.03, 1.32)	1.18 (1.02, 1.35)
Cirrhosis	1.08 (0.88, 1.32)	0.86 (0.68, 1.07)
Peripheral vascular disease	1.2 (0.97, 1.48)	1.15 (0.95, 1.38)
Cancer	0.93 (0.85, 1.02)	0.93 (0.85, 1.01)
Renal stones	0.91 (0.72, 1.17)	0.78 (0.63, 0.97)
Chronic kidney disease	0.99 (0.91, 1.09)	0.94 (0.86, 1.02)
Stage 1	0.88 (0.23, 3.3)	0.51 (0.16, 1.64)
Stage 2	0.89 (0.48, 1.67)	0.83 (0.48, 1.43)
Stage 3	1.07 (0.95, 1.2)	1 (0.9, 1.12)
Stage 4	0.89 (0.74, 1.07)	0.86 (0.72, 1.01)
Stage 5	0.9 (0.74, 1.09)	0.84 (0.7, 1)

**Medications**		
NSAIDs	1.22 (1.1, 1.36)	1.14 (1.04, 1.25)
ACE inhibitors/ARBs	0.99 (0.92, 1.07)	0.91 (0.85, 0.97)
Diuretics		
Thiazide	1.35 (1.18, 1.55)	1.31 (1.16, 1.48)
Loop	1.28 (1.19, 1.37)	1.17 (1.09, 1.27)
Potassium sparing	1.37 (1.22, 1.54)	1.3 (1.16, 1.46)
Other	1.4 (1.04, 1.9)	1.27 (0.97, 1.66)
Proton pump inhibitors	1.26 (1.17, 1.36)	1.19 (1.12, 1.27)
Trimethoprim	1.15 (0.96, 1.37)	1.15 (0.98, 1.35)
Aminoglycosides	1.54 (1.4, 1.69)	1.25 (1.14, 1.37)
Vancomycin	2.26 (1.99, 2.56)	2.33 (2.07, 2.62)
Penicillin	1.53 (1.42, 1.64)	1.39 (1.3, 1.49)
**First AKI stage**		
Stage 1	1.00 (Reference)	1.00 (Reference)
Stage 2	0.93 (0.83, 1.03)	0.92 (0.84, 1.02)
Stage 3	0.93 (0.82, 1.05)	1.01 (0.9, 1.13)
**AKI care plan**		
Not initiated	1.00 (Reference)	1.00 (Reference)
Initiated	1.3 (1.16, 1.45)	1.26 (1.14, 1.39)
**Status**		
Inpatient mortality	0.97 (0.88, 1.07)	0.83 (0.75, 0.9)
COVID-19	1.49 (1.28, 1.72)	1.61 (1.41, 1.84)

[a] Adjusted for age, comorbidities (IHD, hypertension, heart failure, diabetes, liver disease, cirrhosis, PVD, cancer, renal stones, CKD), medications (NSAIDs, ACE inhibitors/ARBs, diuretics [thiazide, loop, potassium sparing, other], proton pump inhibitors, trimethoprim, aminoglycosides, vancomycin, penicillin), first documented AKI stage, AKI-CP and inpatient mortality. RR, rate ratio; NSAID, non-steroidal anti-inflammatory drug; ACE, angiotensin converting enzyme; ARB, angiotensin receptor blocker.

Supplemental Table 7. Characteristics of the respondents to the survey of AKI-CP initiation

		**October 2020**		**July 2021**	
		n	%	n	%
**Total**		53	100	17	100
**By grade**	FY1 (Intern)	15	28.3	4	23.5
	FY2 (First-year resident)	23	43.4	9	52.9
	Specialty trainee (Resident)	14	26.4	3	17.6
	Other	1	1.9	1	5.9
**By specialty**	Medicine	26	49.1	10	58.8
	Surgery	11	20.8	4	23.5
	Emergency Medicine	13	24.5	2	11.8
	Other	3	5.7	1	5.9
